# Ten Issues for Updating in Community-Acquired Pneumonia: An Expert Review

**DOI:** 10.3390/jcm12216864

**Published:** 2023-10-30

**Authors:** Francisco Javier Candel, Miguel Salavert, Miren Basaras, Marcio Borges, Rafael Cantón, Emilia Cercenado, Catian Cilloniz, Ángel Estella, Juan M. García-Lechuz, José Garnacho Montero, Federico Gordo, Agustín Julián-Jiménez, Francisco Javier Martín-Sánchez, Emilio Maseda, Mayra Matesanz, Rosario Menéndez, Manuel Mirón-Rubio, Raúl Ortiz de Lejarazu, Eva Polverino, Pilar Retamar-Gentil, Luis Alberto Ruiz-Iturriaga, Susana Sancho, Leyre Serrano

**Affiliations:** 1 Clinical Microbiology & Infectious Diseases, Transplant Coordination, IdISSC & IML Health Research Institutes, Hospital Clínico Universitario San Carlos, 28040 Madrid, Spain; 2 Infectious Diseases Unit, La Fe (IIS) Health Research Institute, University Hospital La Fe, 46015 Valencia, Spain; 3 Immunology, Microbiology and Parasitology Department, Faculty of Medicine and Nursing, University of País Vasco, 48940 Bizkaia, Spain; miren.basaras@ehu.eus; 4 Multidisciplinary Sepsis Unit, Intensive Medicine Department, University Hospital Son Llàtzer, 07198 Palma de Mallorca, Spain; mborges1967@yahoo.es; 5 Instituto de Investigación Sanitaria Islas Baleares (IDISBA), 07198 Mallorca, Spain; 6 Clinical Microbiology Service, University Hospital Ramón y Cajal, Institute Ramón y Cajal for Health Research (IRYCIS), 28034 Madrid, Spain; rafael.canton@salud.madrid.org; 7 CIBER of Infectious Diseases (CIBERINFEC), National Institute of Health San Carlos III, 28034 Madrid, Spain; pretamar@us.es; 8 Clinical Microbiology & Infectious Diseases Service, University Hospital Gregorio Marañón, 28009 Madrid, Spain; emilia.cercenado@salud.madrid.org; 9 IDIBAPS, CIBERES, 08007 Barcelona, Spain; catiacilloniz@yahoo.com; 10 Faculty of Health Sciences, Continental University, Huancayo 15304, Peru; 11 Intensive Care Unit, INIBiCA, University Hospital of Jerez, Medicine Department, University of Cádiz, 11404 Jerez, Spain; 12 Clinical Microbiology, University Hospital Miguel Servet, 50009 Zaragoza, Spain; jmgarcialechuz@salud.aragon.es; 13 Intensive Care Clinical Unit, Hospital Universitario Virgen Macarena, 41013 Sevilla, Spain; jgarnachom@gmail.com; 14 Intensive Medicine Department, University Hospital of Henares, 28802 Madrid, Spain; fgordo5@gmail.com; 15 Emergency Department, University Hospital Toledo, University of Castilla La Mancha, 45007 Toledo, Spain; agustinj@sescam.jccm.es; 16 Emergency Department, Clinical University Hospital San Carlos, 28040 Madrid, Spain; fjjms@hotmail.com; 17 Anesthesiology Department, Hospital Quirón Salud Valle del Henares, 28850 Madrid, Spain; emilio.maseda@gmail.com; 18 Hospital at Home Unit, Clinic University Hospital San Carlos, 28040 Madrid, Spain; mayra.matesanz@gmail.com; 19 Pneumology Service, La Fe (IIS) Health Research Institute, University Hospital La Fe, 46015 Valencia, Spain; rosmenend@gmail.com; 20 Hospital at Home Service, University of Torrejón, Torrejón de Ardoz, 28006 Madrid, Spain; mmrubio@torrejonsalud.com; 21 National Influenza Center, Clinic University Hospital of Valladolid, University of Valladolid, 47003 Valladolid, Spain; lejarazu@gmail.com; 22 Pneumology Service, Hospital Vall d’Hebron, 08035 Barcelona, Spain; eva.polverino@vhir.org; 23 Vall d’Hebron Institut de Recerca (VHIR), Vall d’Hebron Barcelona Hospital Campus, 08035 Barcelona, Spain; 24 CIBER of Respiratory Diseases (CIBERES), Institute of Health San Carlos III, 28029 Madrid, Spain; 25 Infectious Diseases & Microbiology Clinical Management Unit, University Hospital Virgen Macarena, IBIS, University of Seville, 41013 Sevilla, Spain; 26 Pneumology Service, University Hospital Cruces, 48903 Barakaldo, Spain; lruiziturriaga@gmail.com (L.A.R.-I.); leyre.serranofernandez@osakidetza.eus (L.S.); 27 Faculty of Medicine and Nursing, University of País Vasco, 48940 Bizkaia, Spain; 28 Intensive Medicine Department, University Hospital La Fe, 46015 Valencia, Spain; sanchosus@gmail.com

**Keywords:** community acquired pneumonia, aetiology, radiologic findings, management, readmission, hospital at home, therapeutic failure, vaccination

## Abstract

Community-acquired pneumonia represents the third-highest cause of mortality in industrialized countries and the first due to infection. Although guidelines for the approach to this infection model are widely implemented in international health schemes, information continually emerges that generates controversy or requires updating its management. This paper reviews the most important issues in the approach to this process, such as an aetiologic update using new molecular platforms or imaging techniques, including the diagnostic stewardship in different clinical settings. It also reviews both the Intensive Care Unit admission criteria and those of clinical stability to discharge. An update in antibiotic, in oxygen, or steroidal therapy is presented. It also analyzes the management out-of-hospital in CAP requiring hospitalization, the main factors for readmission, and an approach to therapeutic failure or rescue. Finally, the main strategies for prevention and vaccination in both immunocompetent and immunocompromised hosts are reviewed.

## 1. Introduction

Community-acquired pneumonia (CAP) represents the most important cause of mortality due to infection in industrialized countries. Excluding the impact of COVID-19, it has an incidence of 1.2 to 2.4 cases per 1000 adults in Europe–USA. These differences have been attributed to the higher rate of pneumococcal vaccination in Europe. At extreme ages (under 5 and over 65–70), the incidence increases [1].

Although the guidelines for the approach to this model of infection are widely implemented in international health schemes, there is variability in the diagnostic-therapeutic management, with differences in admission rates, the achievement of microbiological diagnosis, request for complementary studies, the choice of antimicrobial regimen, or the diversity of care applied. In addition, information that generates controversy or requires an update in its management is constantly emerging.

The aim of the present paper was to review the ten topics that have undergone the greatest updates in community-acquired pneumonia such the implementation of new molecular platforms to discern the etiology, the diagnostic guidance in different clinical settings, the applicability of imaging techniques, or the criteria for admission to the Intensive Care Unit and clinical stability at discharge. Thus, we include an update on antimicrobial, oxygen, or steroid therapy. Other topics reviewed in this document are out-of-hospital management of CAP requiring hospitalization, main factors for readmission, and the approach to therapeutic failure or rescue. Finally, we reviewed the main strategies for prevention and vaccination in both immunocompetent and immunocompromised hosts.

## 2. Material and Methods

Design. From the Study Group of Infection in the Critically Ill Patient of the Spanish Society of Infectious Diseases and Clinical Microbiology (GEIPC-SEIMC), in January 2023 experts were requested from all scientific societies attending community-acquired pneumonia listed in the document’s affiliation, grouping two authors per topic. They were asked for a narrative review. Search strategy. Between January and June 2023, the experts performed a bibliographic search of their corresponding topics in PubMed (http://www.ncbi.nlm.nih.gov/pubmed/, accessed on 2 September 2023), Embase (http://www.elsevier.com/online-tools/embase/, accessed on 2 September 2023), and Scopus (http://www.elsevier.com/online-tools/scopus, accessed on 2 September 2023), choosing those that, in their experience, were most relevant or most current, up to a maximum number of 15 references, excluding the rest. Drafting. In June 2023, the texts were received from the experts, with a limit of two pages per topic. Some of them, due to the content of the assigned topic, were also asked to include a figure or a table. Between June and July 2023, the coordinators integrated the texts. Revision. Between July and August 2023, all the experts had the opportunity to read the complete text and raise objections and changes.

## 3. Results

### 3.1. Aetiologic Update on CAP

The microbiological diagnosis of community-acquired pneumonia (CAP), as any other infectious process, involves the identification or detection of the causative agent and information regarding the most appropriate treatment based on in vitro susceptibility testing studies, particularly for bacterial pathogens. Traditionally, the aetiology of CAP has been established with studies limited by time and with laboratory techniques restricted to bacterial culture, immunological assays based on the detection of antibodies or antigens, and, occasionally, with molecular methods targeted to specific pathogens or just considering a limited number of them [1,2]. However, guidelines from different societies do not always recommend the use of the same techniques and respiratory samples [3]. Thus, the picture that has been obtained has sometimes been partial, limited to specific groups of population (paediatric, adult, elderly, immunocompromised patients, etc.), during periods characterized by seasonal pathogens, and almost always limited to patients requiring hospital admission, either on clinical wards or at critical care units [4]. From a microbiological point of view, causative organisms were not found in a high proportion of patients with CAP (up to 70%) [1]. Possible reasons that may explain this insufficient information of the aetiology of CAP might include the diversity of respiratory samples used from the lower respiratory tract (sputum, bronchial aspirates, brochoalveolar lavages, protected telescope catheter samples, etc.), difficulties in obtaining these samples, the effect of antibiotic use prior to sample collection, low sensitivity of some of the diagnostic tests, and the involvement of viruses that have not been frequently investigated in CAP [5].

This traditional picture could have been changed due to the progressive introduction of the so-called syndromic platforms or syndromic panels in laboratory CAP diagnosis [6]. They are generally based on real-time PCR techniques that include a relevant number of pathogens as targets (bacteria, viruses and/or fungi) and whose use improves the identification of the microorganism causing CAP, including the detection of co-infections and genes associated with resistance to antimicrobial agents. However, they also complicate the interpretation of the results by finding microorganisms with doubtful pathogenicity, coinfections, and resistance genes that are occasionally not expressed with discrepancies found with phenotypic susceptibility studies [7]. Moreover, these panels have different designs covering different pathogens from different manufacturers, causing complicated comparison of data from the studies and between laboratories [6,8]. Nevertheless, the COVID-19 pandemic has led to a positive introduction of these platforms and syndromic panels in the routine of clinical microbiology laboratories, particularly for most critical patients [7]. The introduction of these syndromic platforms has led to discussion about the role of viruses in CAP [5]. Despite this, it is currently recommended to use a multiplex PCR panel to detect epidemic respiratory viruses in patients hospitalized with CAP in order to discontinue antibacterial treatment in case of positivity and no evidence of coexisting bacterial infection or clinical deterioration [9].

In a pre-COVID-19 pandemic study, Gilbert et al. demonstrated the increase in pathogen detection in CAP, both bacterial and virus-related, using syndromic panels when compared with a bundle of conventional methods (66.4% vs. 75.5% and 40.5% vs. 60.9%, respectively) [10]. Interestingly, some classical pathogens decreased (e.g., *Streptococcus pneumoniae*), while others increased with the use of these panels (e.g., *Haemophilus influenzae*, Rhinovirus, and Influenza virus). This was corroborated in a recent published review that depicts how the introduction of different techniques over time has had an impact on the understanding of the involvement of classical pathogens on CAP [2]. This review highlights a decreasing role of *S. pneumoniae* as the main causative pathogen, emphasizing other ones such as *H. influenzae, Staphylococcus aureus, Moraxella catarrhalis*, Enterobacterales, *Pseudomonas aeruginosa*, as well as the so-called atypical pathogens and, interestingly, commensal bacteria, also recognized as “normal respiratory microbiota” [2]. Moreover, the impact of COVID-19 in the presence of pathogens in CAP has also been investigated. Table 1 includes pathogen identification in different periods and with the use of different technologies [2,11,12]. In the future, the use of metagenomics techniques will enlarge the knowledge of aetiology of CAP, depicting the entire microbiome, including anaerobes, which are rarely included in publications [2]. Due to the complexity of obtaining information in the microbiome characterization, bioinformatics analysis coupled with artificial intelligence will be necessary to understand the role of different microorganism populations (microbiome signature) and interaction with the host [13]. In addition, metabolomics and metatranscriptomic analysis will be necessary to underscore potential biomarkers that alert evolution of patients or become useful for personalized medical or lifestyle interventions.

Currently, the cost-effectiveness of the syndromic panels remains to be fully validated in terms of price and usefulness in the management of the patients and impact of the information [6]. Different studies demonstrated reduction in time to results and impact in antimicrobial stewardship actions, including early adaptation of antimicrobial therapy [14,15,16].

Regarding phenotypic in vitro susceptibility studies, the most important novelties correspond to the change in the criteria introduced by the European Committee of Antimicrobial Susceptibility testing (EUCAST) [17] in 2019, which affects the clinical susceptibility categories resulting from the interpretation of the antibiogram with the established breakpoints [18]. The most relevant change is in the “intermediate category”, which now becomes category “I”, with a meaning of “susceptible, increased exposure”. There is also a minor change in the “susceptible” (S) category, which is now defined as “susceptible, standard dose”, with no change in the consideration of the resistant (R) category. In *S. pneumoniae*, this change has not been drastic for beta-lactam antibiotics. EUCAST already established different categories for penicillin according to the dose used; microorganisms with certain increase in MIC values are considered susceptible as long as the dose used is higher (EUCAST Breakpoint table-2023). For some quinolones and *S. pneumoniae*, such as levofloxacin, the “S” category disappears and the entire wild-type population (microorganisms without acquired resistance mechanisms) is considered “I” (susceptible, increased exposure) to favor the adequate prescription of the antimicrobial by using the right high does (0.5 g × 2 oral or 0.5 g × 2 iv) necessary to obtain an adequate outcome and not using the standard dose (0.5 g × 1 oral or 0.5 g × 1 iv), which is insufficient to ensure this success. The same situation occurs with *H. influenzae* and amoxicillin or amoxicillin-clavulanate when used orally; the “S” category disappears, and the entire wild-type population is considered “I” (susceptible, increased exposure).

### 3.2. Aetiologic Approach to CAP Using Imaging Techniques

CAP is defined as the presence of new pulmonary infiltrate on chest X-ray or other chest image techniques together with acute signs and symptoms suggestive of lower respiratory tract infection. The primary role of imaging examinations in CAP is to confirm the diagnosis of pneumonia. However, sometimes the radiological pattern may allow us to make an aetiologic approach of CAP at the time of diagnosis. From a practical point of view, we currently have three commonly used imaging techniques for the diagnosis of this entity and its potential associated complications.

The chest X-ray is the most used image method to make the CAP diagnosis; it is low-cost and fast [19]. However, there are a small number of immunocompetent patients with CAP who had radiological evidence of pneumonia on computed tomography but not on a concurrent chest radiograph. Despite having less inflammatory charge, these patients have pathogens and clinical outcomes similar to those who had signs of pneumonia on a chest radiography [20,21]. Depending on the location and the type of the infiltrate, it is possible to distinguish between viral, typical bacterial, or atypical bacterial CAP (Table 2). Typical CAP patterns on imaging examinations are consolidation (alveolar/lobar pneumonia), peribronchial nodules (bronchopneumonia), and ground-glass opacity (GGO). In fact, many pathogens can cause pneumonia with more than one pattern [22].

Consolidation predominant pneumonia is referred to as alveolar pneumonia and it usually appears in typical bacterial pneumonia such as that caused by *S. pneumoniae*. It is characterized in histology by the alveolar spaces being filled with an inflammatory exudate, with little or no tissue damage [23]. Radiographically, it shows a nonsegmental, homogenous consolidation involving predominantly or exclusively one lobe with or without visible air bronchogram. Peribronchial nodules predominant pattern occurs when infectious organisms deposited on the epithelium of the bronchi produce acute bronchial inflammation with epithelial ulcerations and fibrinopurulent exudate formation; CAP with this pattern is called bronchopneumonia [23]. The bacteria most often involved are *S. aureus*, *Haemophilus influenzae*, *P. aeruginosa*, anaerobes, and some species of fungus, especially *Aspergillus*. In ground-glass opacity predominant pattern, the initial damage is directed toward the mucosa of the bronchioles, and, later, the peribronchial tissue and interlobular septa become edematous and infiltrated with inflammatory cells [23]. It is usually bilateral and associated with viruses, *Mycoplasma pneumoniae*, and *Pneumocystis jirovecii* CAP.

Computed tomography (*CT*) scan might be helpful for diagnosing CAP, especially when the result of the chest X-ray is inconclusive, as it has higher sensitivity, and it may help to perform a differential diagnosis with other illnesses as lung cancer, pulmonary edema, or exacerbation of chronic obstructive pulmonary disease; it can also help to fidentify CAP complications [24]. On the other hand, it is more expensive, involves higher exposure to radiation, and is not as usually available as X-ray. Claessens et al. [25] explored the impact of systematic early chest CT scan on diagnosis in patients visiting the emergency department. After analyzing 319 patients with suspected CAP, CT scanning revealed a parenchymal infiltrate in 33% of the patients without infiltrate on chest radiograph and excluded CAP in 29.8% of the 188 with parenchymal infiltrate on X-ray. CT scanning modified classification in 58.6% (95% confidence interval, 53:2–64).

General indications of CT scanning for CAP include severe or complex pneumonia, pneumonia in immunocompromised hosts, pneumonia intractable to antibiotics, recurrent or non-resolving pneumonia, and patients with clinical suspicion of pneumonia but normal or questionable chest radiographic findings [26]. The typical findings from CT scanning in CAP patients are ground-glass opacities, airspace nodules, consolidation, air bronchograms, and bronchial wall thickening (Table 2) [27]. Ground-glass opacities are defined as a localized increase in lung attenuation that allows visualization of vascular structures coursing through the affected region. As previously discussed, it may be attributable to infection caused by viruses, *Pneumocystis jirovecii*, CMV, and *Mycoplasma* spp. A “tree-in-bud” pattern reflects the presence of bronchioles filled with mucus or inflammatory material, resulting in centrilobular tubular, branching, or nodular structures. A variety of bacterial, mycobacterial, fungal, and viral pathogens may cause bronchogenic dissemination and bronchiolar impaction by mucus or pus, resulting in a tree-in-bud pattern. Focal consolidation, defined as a localized increase in lung attenuation that does not allow visualization of vascular structures coursing through the affected region, may be seen in association with bacterial, fungal, and viral infections. Bacterial pneumonia is the most common cause of pulmonary consolidation [28].

In recent years, point-of care lung ultrasound is gaining special importance in the diagnosis and complication management of patients with CAP. Lung ultrasound has both a higher sensitivity and specificity than chest X-ray in detecting consolidation but requires clinician experience [29]. One of the benefits of the lung ultrasound is that it is completed at the bedside and results are available to the clinician in real time to aid in diagnosis and decision-making. Clinicians can perform serial examinations to monitor disease progression and treatment response. An important limitation of this technique is the experience of the clinician who performs and interprets the examination. On ultrasound, finding either consolidation pattern or focal interstitial syndrome had the highest sensitivity (0.96); finding isolated focal interstitial syndrome or isolated anterior consolidation had the best specificity (0.97) for CAP diagnosis [19]. Mearelli et al. [30], in a recent study of 420 patients with low respiratory tract infection, demonstrated that the AUC for diagnosing CAP by lung ultrasound was significantly higher than that for diagnosing CAP by chest X-ray (0.93 and 0.71, respectively; *p* < 0.001). This study distinguished two patterns: pattern 1 (one or more subpleural consolidations with or without one or more areas of alveolar-interstitial syndrome) and pattern 2 (one or more areas of alveolar-interstitial syndrome). Pattern 1 was significantly associated with bacteria CAP (*p* < 0.001) and more linked to clinical deterioration and 30-day mortality. Pattern 2 ruled out mortality with a negative predictive value of 95% (95% CI, 86–98%). Given this, lung ultrasound could help clinicians to predict the need for microbiological cultures or empirical antibacterial therapy. Table 2 shows different lung ultrasound patterns depending on CAP etiology.

### 3.3. Diagnostic Stewardship in Different Clinical Settings

Rapid microbiological diagnosis is essential for a proper and targeted treatment. In the case of community-acquired pneumonia (CAP), early identification of the causative pathogen is crucial to guide antibiotic therapy, prevent the emergence of antimicrobial resistance, and reduce avoidable drug adverse effects [31]. However, approximately 30–50% of all CAP cases lack an etiologic diagnosis and, in most cases, the treatment is empirical [32,33]. New diagnosis tools such as point-of-care (POC) tests or rapid respiratory syndromic panels may have a positive effect on microbiological diagnosis of patients with CAP, although its impact on outcomes is controversial [32]. Therefore, diagnostic recommendations in patients presenting with CAP may differ according to patient setting, severity of presentation, and previous immunosuppression (Table 3).

In community-care settings, including primary care, outpatient clinic, emergency department, and long-term care facilities, the prescription of antibiotics is often taken without availability of diagnostic tests. In these circumstances, POC tests could be helpful in differentiating bacterial and viral acute community-acquired lower respiratory tract infections, facilitating antimicrobial stewardship in the community [31,34]. However, although rapid antigen-based diagnostic tests for influenza, respiratory syncytial virus, human metapneumovirus, and *Streptococcus pneumoniae* show a high specificity (>80%), all have a sensitivity ranging from 49% to 84%, which is suboptimal. Therefore, when positive, they can be used to confirm the diagnosis, but negative results are not reliable due to its high false-negative rates [34]. Molecular tests for detecting all these pathogens are accurate diagnostic tools, but their use at POC in community-care settings needs to be explored [37,38]. On the other hand, the determination of C-reactive protein and procalcitonin has been shown to reduce antibiotic prescribing in primary care by helping to reduce clinical uncertainty. However, its use has limited utility in antimicrobial initiation in patients with CAP and is not recommended [31,39].

In patients attending the emergency department with suspected CAP, the usefulness of routine microbiological testing to rationalize antibiotic use and improve clinical outcomes is under debate. Among patients hospitalized with CAP, the rate of positive blood cultures is low, ranging from 4.7 to 16%, and the diagnostic yield of sputum cultures is <50% [40,41]. Regarding the diagnosis of bacterial infections, in adults with severe CAP managed in the hospital setting and in immunocompromised patients with CAP, it is recommended to obtain blood cultures at the time of diagnosis and perform a pre-treatment Gram-stain and culture of respiratory secretions. Gram-stain and cultures are also recommended in patients who are being empirically treated for methicillin-resistant *Staphylococcus aureus* (MRSA) or *Pseudomonas aeruginosa*, those who were previously infected with these organisms, and in patients previously hospitalized that received parenteral antibiotics in the last 90 days [31,40,41]. In this scenario, nares screening for MRSA had a high specificity and negative predictive value for ruling out MRSA pneumonia [35,42].

Urinary antigen tests (UAT) for *S. pneumoniae* and *L. pneumophila* are simple, non-invasive, rapid, non-culture-based diagnostic tests that detect antigens from pathogens excreted in the urine and are unaffected by prior antibiotic administration. They show a high specificity (>90%) and moderate sensitivity (<80%) [36]. Data from a meta-analysis [34] showed an overall sensitivity of 70% (95%CI 60–79%) and specificity of 83% (95% CI 63–93%). Importantly, most of the commercially available *Legionella* spp. Tests are only able to detect the most common subtype, *L. pneumophila* serogroup 1, which may lead to missed diagnoses. Therefore, they should be used in combination with other diagnostic tests. In adults with severe CAP, *Legionella* spp. Culture on selective media or the use of nucleic acid amplification testing from respiratory samples is recommended [31,35,36]. Despite their potential usefulness, current American Thoracic Society and Infectious Diseases Society of America (ATS/IDSA) guidelines do not recommend the routine use of urinary antigen tests, except in patients with severe CAP and in those with epidemiological risk factors (as a potential outbreak) [31] or if the patient has hyponatremia, fever, headache, diarrhea, or a recent travel. The indications for UAT for *S. pneumoniae* include ICU admission, failure of outpatient antibiotic therapy, active alcohol abuse, pleural effusion, leukopenia, chronic liver disease, and asplenia [35,36].

Over the last few years, commercially available nucleic acid amplification technologies have emerged as the diagnostic tools of choice for respiratory pathogens, particularly viruses, but also for the detection of difficult to grow bacteria and some resistance genes. These molecular assays should be used for the detection of influenza viruses when these are circulating in the community and for the detection of SARS-CoV-2 [31,33,34]. During periods of low influenza activity, testing must be considered, especially in immunocompromised patients. It is also necessary to emphasize that this testing recommendation has both therapeutic and infection-control implications in the hospital setting [31]. At present, the wide availability of commercialized rapid respiratory syndromic panels has shortened the diagnostic turnaround time to one to five hours and is less likely to be affected by prior antibiotic administration [33,38]. The benefits of these rapid syndromic panels include their high sensitivity, specificity, and negative predictive value when used in conjunction with expert interpretation, but they should not replace conventional culture and antimicrobial susceptibility testing. Their limitations include lack of detection for off-target pathogens, lack of full susceptibility information, cost, and false-positive results due to the detection of nucleic acids from dead pathogens not currently causing active infection [38,43]. Current IDSA guidelines for CAP do not address molecular testing for bacterial pathogens as it remains unclear which patients would benefit most from their use [31,43,44,45] (Table 3).

### 3.4. Intensive Care Unit Admission Criteria and Clinical Stability (ICU-Ward-Discharge)

More than 30% of patients with community-acquired pneumonia (CAP) admitted to the hospital require admission to the ICU, with mortality in these cases being greater than 50%. Delayed ICU admission in patients with CAP has been shown to increase mortality, especially in elderly patients, patients with multiple comorbidities, and immunocompromised patients [1,46,47]. Timely appropriate antibiotic therapy and delay in ICU admission are two important factors that contribute to the better or worse outcome in CAP patients [48,49,50].

Since there is no universal definition of severe pneumonia, multiple severity scales have been developed to identify those patients who would benefit from ICU admission. The two most widely used scores were the pneumonia severity index-PSI and the CURB-65, which were validated to predict 30-day mortality in CAP patients but had limitations to predict the necessity of ICU admission. Prediction mortality is different from predict severity, and the main reasons are the strong influence of age and the lack of markers or criteria for organ dysfunction, which represent a severity criterion in pneumonia. This is the main reason that other prognostic scores such as the SMART-COP [51] and the American Thoracic Society (ATS)/Infectious Disease Society of America (IDSA) criteria [52] were developed to identify severe CAP cases that required ICU admission. A limitation of SMART-COP is the complexity for the variables included in the score and the different cut-off that may limit their use as daily routine [53]. Despite this, there is not a clear definition for severe CAP. Several studies have validated the ATS/IDSA criteria demonstrating that these criteria had the highest predict prognosis for ICU admission in patients with severe CAP [54,55,56,57].

According to the ATS/IDSA criteria, the need for mechanical ventilation with endotracheal intubation or the presence of septic shock requiring receipt of vasopressors are major criteria for ICU admission. There are nine minor criteria that include respiratory rate (≥30 breaths/min), PaO_2_/FiO_2_ ratio (≤250), multilobar infiltrates, confusion/disorientation, uremia (BUN level, ≥20 mg/dL), leukopenia (<4000 white blood cells/mm^3^), thrombocytopenia (platelet count, <100,000 cells/mm^3^), hypothermia (core temperature, <36 °C), and hypotension requiring aggressive fluid resuscitation (Table 4). The presence of three of the nine minor criteria indicate that a patient that will require ICU admission.

An interventional trial by Lim et al. [56] demonstrated the accuracy of the minor ATS/IDSA criteria in improving CAP outcomes. They identified severe cases of pneumonia early in the ED and initiated early intervention, such as initiation of antibiotic treatment within 3 h, rapid initiation of invasive mechanical ventilation in case of respiratory failure, and early interventions in case of patients presenting with hypotension (fluid resuscitation) or shock (vasopressors). The study reported a decrease in the mortality rate to 6% (intervention group) vs. 24% (control group), *p* < 0.001. They also reported a decrease in the rate of ICU admission from 53% to 39%, *p* < 0.008, respectively, and a decrease in the inappropriate delay ICU admission from 32% to 15%, *p* < 0.001, respectively.

The clinical evidence showed that the ATS/IDSA criteria are the most accurate for ICU admission and improved the prediction of mortality in CAP. Pneumonia is a systemic disease and in severe cases there is a frequent development of organ dysfunction, especially in the first 48 h to 72 h, so a timely management measures such as early antibiotic therapy, prompt intubation in case of respiratory failure, and early intervention in case of shock are measures associated with better outcomes and decrease mortality [58].

Clinical stability in patients who respond to treatment is achieved during the first three days. The time to clinical stability is an important variable in the management of CAP patients that could impact outcomes after hospital discharge. The use of biomarkers (such as procalcitonin (PCT) and C-reactive protein (CRP)), which bring information about inflammation, response to the infection, and response to the antibiotic therapy are associated with the clinical stability criteria (temperature ≤ 37.2 °C, heart rate ≤ 100 beats/min, respiratory rate ≤ 24 breaths/min, systolic blood pressure ≥ 90 mmHg, and oxygen saturation ≥90% or arterial oxygen tension ≥60 mmHg when the patient was not receiving supplemental oxygen), may improve the safe identification of patients that reach clinical stability. For patients on home oxygen therapy, stability was considered to be achieved when their oxygen needs were the same as those before admission) [59].

### 3.5. Antibiotic Treatment Update

Community acquired pneumonia is defined as an acute infection of the pulmonary parenchyma acquired outside of health care settings. It deserves special attention because of immunological worn-out after the SARS-CoV-2 pandemic [60].

Primary care management of patients with a lower respiratory tract infection or community pneumonia is based on the following steps: (1) Identify the patients who need to be treated in a hospital Emergency Department, that is, establish the severity of the disease; (2) Establish which patients can benefit from specific antibiotic treatment; (3) Decide when rapid diagnostic microbiological tests are indicated, including influenza, VRS, and COVID tests; and (4) prescribe the most effective antibiotic treatment. The current guidelines published by scientific societies for the antibiotic treatment of patients with CAP recommend to choose initial treatment depending on the pneumonia setting: outpatient, hospitalization in conventional ward, or in ICU. The rational for the stratification relies on both the initial severity and the most probable causal microorganisms. The goal is to adequately cover the potential etiology to avoid inappropriate treatment that is related to mortality mainly in those with more severe episodes [1].

In outpatients, the current recommendations consider that quinolones in monotherapy or the combination of a beta-lactam and a macrolide are the regimens with adequate spectrum for typical and atypical intracellular bacteria. In some countries, especially those in the north of Europe, monotherapy with a betalactam alone (amoxicillin or amoxiclav are preferred, may still be effective in non-severe cases [61]. However, this is not the case in the south of Europe, where the combination betalactam plus macrolide leads protocols and national guidelines [62]. There is a controversy about the necessity of adding a macrolide to cover atypical microorganisms instead of using beta-lactam in monotherapy [63] because its superiority has not yet been definitively established. However, Asadi et al. [64] have reported lower mortality and admission requirements in those treated with macrolide combinations (0.2% vs. 3%). The effect of macrolide is also found in patients with drug-resistant *S. pneumoniae* [65]. Moreover, in a study recently published in Chest, Bai et al. reviewed data from 23,512 consecutive patients admitted to 19 hospitals in Canada for community-acquired pneumonia between 2015 and 2021 [66]. Patients were treated with one of four initial antibiotic regimens: beta-lactam plus macrolide (9340 p), beta-lactam alone (9146 p), respiratory fluoroquinolone (4510 p), or beta-lactam plus doxycycline (516 p). Adjusted in-hospital mortality was not significantly different between beta-lactam plus macrolide and fluoroquinolone or beta-lactam plus doxycycline, but the 1.5% difference seen with beta-lactam alone indicated a “small but clinically important difference”; patients treated with beta-lactam alone also had a longer time to hospital discharge.

In patients with CAP who require hospital admission, treatment with the combination of a beta-lactam and a macrolide or a quinolone is recommended to cover the most frequent causal microorganisms. A meta-analysis of 28 observational studies [67] with 9850 patients diagnosed with severe CAP demonstrated a 3% reduction in mortality (relative risk, RR 0.82, *p* = 0.02) when a macrolide was included in the antibiotic regimen compared to other antibiotics. Its proven benefit may be due to its anti-inflammatory effect in severe patients. Ceccato A et al. [68] demonstrated that, in patients admitted with pneumococcal CAP and a high systemic inflammatory response, the combination of a beta-lactam with a macrolide significantly reduced mortality. However, in a small percentage there is a possibility of multi-resistant or difficult to treat microorganisms. Therefore, a strategy to rule out or suspect *Pseudomonas aeruginosa*, extended-spectrum beta-lactamase-producing *Enterobacteriaceae*, and methicillin-resistant *Staphylococcus aureus* has been proposed using a new and validated score: the PES score [69]. The empirical antibiotic should consider the PES etiology if score punctuation is ≥5 (70% sensitivity). Similarly, new ATS/IDSA recommendations consider it advisable to discard *S. aureus* or *Pseudomonas* spp. etiology in patients with locally validated risk factors and in those requiring hospitalization in ward or the ICU. In the ATS/IDSA guidelines, antiinfluenza treatment with oseltamivir is recommended to be prescribed for adults with CAP who test positive for influenza, independent of duration of illness before diagnosis [31]. The main initial recommendations related to antimicrobial therapy are shown in Table 5.

The optimal duration of antibiotic treatment in CAP is not well established, and there are discrepancies between the different guidelines published to date. Nevertheless, there is agreement that it should be individualized and based on clinical stability criteria, with a minimum of 5 days, and it can be suspended after 48 h of absence of fever (temperature < 37.8 °C) and without more than one sign of clinical instability (pressure systolic blood pressure < 90 mmHg, heart rate > 100 beats/min, respiratory rate > 24/min, <90% room air) [62]. The optimal duration of therapy in necrotizing pneumonia, lung abscess, complicated pleural effusion, or suspicion of unusual microorganisms (*P. aeruginosa*, *S. aureus*, anaerobes) is not well known, and it could be prolonged in those circumstances.

There are three keys for selecting the appropriate oral antibiotic for respiratory infections. First is effectiveness, with the aim of achieving maximum microbiological eradication using the antibiotic with the narrowest spectrum for the most isolated microorganisms in this type of infection. Second is safety, minimizing the probability of adverse effects related to the antibiotic, especially the most serious ones, and finally, the microbiota, trying to have the least possible impact on it, since the loss of its diversity leads to greater vulnerability to infection and resistance selection [70]. The current policy is reducing the burden of antibiotics and their adverse effects while ensuring no negative impact on outcomes. The negative effects of prolonging the duration of antibiotic administration are numerous: *Clostridioides difficile* infection, MDR, adverse effects, and others. In a recent meta-analysis [71] that included 15 randomized and controlled clinical trials with 2796 patients with mild-moderate CAP, no differences were observed in the efficacy of short antibiotic regimens of <7 days vs. ≥7 days. Two meta-analyses [72,73] included several clinical trials in adults with CAP comparing short regimens (less than 7 days) and long regimens (≥7 days); no differences were detected in the clinical cure rate, mortality, and adverse effects between short and long regimens. In the last reported meta-analysis [73], concerning a sub-analysis of patients with severe pneumonia, mortality continued to be lower in the short-course group (2.2% vs. 4.7%). Despite a significant difference in treatment duration (median 5 days and 10 days, respectively, *p* < 0.001), the clinical cure rates at 10 and 30 days were similar.

Biomarkers may be useful to safely reduce and/or personalize treatment duration. The use of procalcitonin (PCT) has shown good potential to reduce antibiotic treatment without adverse effects for patients. In the PRORATA study [74] carried out in critically ill patients, an algorithm was implemented to discontinue the administration of antibiotics after a reduction in PCT of at least 80% or with values < 0.5 µg. Patients in the PCT group had more antibiotic-free days, with an absolute difference of 2.7 days (95% CI 1.4–4.1, *p* < 0.0001) compared to the control group. In another clinical trial with similar characteristics, the median treatment with antibiotics was five days in the PCT group, compared to seven days in the control group. In this study, a significant difference in mortality was observed in favor of the PTC group, both in the intention-to-treat and in the per-protocol population analyses [75].

Initial AB selection is key to provide a narrow, guideline-recommended spectrum antimicrobial that results in better outcome and survival. Scientific guidelines provide recommendations for selection that mainly depends on AB setting and risk factors for MR microorganisms. The choice between these options requires a risk–benefit assessment for each individual patient, weighing local epidemiological data against individual risk factors such as documented beta-lactam or macrolide allergy. For antibiotic treatment duration, infection and clinical parameters indicating clinical stability are decisive in guiding antibiotic duration. That is, when clinical stability is achieved, and duration of antibiotic therapy is between 5–7 days, no biomarkers are needed. PCT guided duration could add benefit in those with prolonged regimens, with most severe episodes and/or complications.

### 3.6. Oxygen and Steroidal Therapy in CAP

Oxygen therapy. Pneumonia is a frequent cause of hypoxemia and is one of the most frequent causes of acute respiratory distress syndrome (ARDS) [76]. The SARS-CoV2 pandemic has also highlighted the potential severity of respiratory failure in viral pneumonia and has generated the proliferation of many oxygen therapy devices [77,78]. Hypoxemia is the consequence of an imbalance in the ventilation/perfusion ratio related to flooding or collapse of the alveoli and local inflammatory phenomena caused by the etiologic agent. This situation results in a shunt effect and secondary hypoxic pulmonary vasoconstriction that diminishes or makes gas exchange impossible. The clinical impact of hypoxemia will increase with age, frailty, or comorbidities and will depend on the lung surface area affected [79].

Hypoxemia is an indication for respiratory support. There are different forms of supplemental oxygen administration depending on clinical oxygen requirements, ranging from low-flow oxygen therapy to extracorporeal oxygenation systems (ECMO). The treatment of hypoxemia must be balanced with the risk of excessive oxygen intake and matched to the clinical response and the rest of the therapeutic strategy [80,81]. In view of this variability in the presentation and therapeutic possibilities, there are several aspects, included in the clinical practice guidelines, aimed at personalizing this support [82,83,84,85]: (i) Monitoring of the oxygen level achieved by SpO_2_ should be maintained. The target to achieve is an SpO_2_ between 94% and 98%, which can be decreased to a range between 88% and 92% in patients with known chronic respiratory failure or patients with ARDS requiring very high levels of O_2_ supply (over 70%); (ii) The use of high or low flow systems should be decided according to the level of oxygenation achieved and the patient’s work of breathing. There is controversy about which system to use [86,87]. In any case, adequate monitoring should be maintained, with the ROX index being useful [88]. Similarly, consideration of escalation from respiratory support to mechanical ventilation should be based on the appearance of work of breathing and respiratory acidosis; (iii) In case of need for mechanical ventilation, a protective strategy should be maintained to avoid lung injury associated with mechanical ventilation; and (iv) escalation to ECMO should be performed in centers with experience [89] and high volume with Extracorporeal Life Support Organization (ELSO) criteria.

Patients with high oxygen intake should have adequate monitoring and surveillance, and the possibility of integrating the amount of oxygen administered into systems for early detection of clinical deterioration, associated with the use of SpO_2_ and respiratory rate, has been suggested.

Corticosteroid therapy. Corticosteroid therapy has been the subject of debate for several years. The results of randomized clinical trials are variable when mortality is measured [90,91,92,93,94]. In patients admitted to the hospital ward, benefits have been described in other objectives, such as avoiding admission to the ICU or reducing therapeutic failure, but without benefits in reducing mortality [94]. The variability in the benefits of corticosteroid treatment depends on the severity of the pneumonia, the patient’s comorbidity, and the intensity of the inflammatory response related to its etiology. In patients admitted with severe pneumonia, no mortality benefits have been found, even adjusting for severity by the Pneumonia Severity Score Index (PSI) [95]. Although a recent meta-analysis focused only on clinical trials on severe community pneumonia points to the benefits of corticosteroids, it still raises doubts since the criteria for severity of pneumonia were not uniform and the pooled evidence remains inconclusive [96]. Another recent study carried out in 31 French ICUs [97] shows a benefit in terms of mortality with the use of hydrocortisone vs. placebo; however, a significant number of patients were excluded due to lack of knowledge of the severity criteria and a very low percentage were on vasoactive support, which suggests a patient closer to a patient admitted to the hospital ward than to a critical patient. The consequence is the need for individualization in steroid therapy, which has achieved promising results in the treatment of respiratory distress [98]; this does not benefit all clinical profiles in the case of COVID [99] and could have a negative impact on the immunologic control of the infection.

The main undesirable effects related to the use of steroids in severe pneumonia have been complications derived from increased glycaemia [92,97], which may favor hemorrhagic complications, increased infections, or readmissions [100].

In relation to etiology, there are differences between the recommendations for steroid treatment in viral pneumonias. Thus, their use is not recommended in the treatment of influenza [101], but they are part of the therapeutic strategy among those caused by SARS-CoV-2 [102]. Among bacterial pneumonia, scientific societies do not routinely recommend the administration of corticosteroids in the treatment of community-acquired pneumonia, limiting their use to those who present refractory septic shock [31] or have another pathology indicating such treatment. As the impact that this recent evidence [97] may have on future guidelines, we consider it prudent to individualize steroid treatment according to etiology, severity, and comorbidity.

### 3.7. Out-of-Hospital Management of CAP Requiring Hospitalization

The Pneumonia Severity Index (PSI) and CURB-65 [103,104] severity and mortality risk scales have been used as decision-making tools for the management of community-acquired pneumonia (CAP) patients. However, there are clinical situations where these scales do not adequately identify whether treatment should be on an outpatient or inpatient basis. Up to one third of hospitalized patients with a low-risk PSI (categories I to III) may present a contraindication to outpatient treatment [105], while some patients with a high-risk category (PSI IV and V) could avoid hospital admission with adequate resources [106]. Outpatient management in hospital at home (HaH) units may be an alternative for patients with CAP where severity scales do not adequately discriminate the most appropriate treatment site.

HaH is model of care that allows patients to be cared for at their place of residence, who would otherwise have to remain hospitalized. HaH has proven to be an effective and safe alternative for a variety of acute illnesses [107]. In CAP patients, HaH teams provide parenteral antimicrobial therapy, oxygen therapy, as well as iv corticotherapy and nebulised bronchodilator therapy when associated bronchospasm is present. In addition, it is possible to monitor and treat co-morbidities, obtain samples for laboratory analysis and microbiological studies, and manage imaging tests, as with hospitalized patient.

Currently, there are no universally accepted criteria for admission to HaH of patients with CAP in their admission avoidance scheme. Sometimes, the final decision is based on the clinical judgement of HaH professionals, which poses a risk of variability in clinical practice. Traditionally, pleural effusion, multilobar pneumonia, and respiratory failure have been considered exclusion criteria for admission to HaH from hospital EDs. However, even these situations require individualized assessment. A small pleural effusion or bilobar pneumonia in a young patient with no risk factors and no evidence of respiratory compromise can be managed in HaH with the same guarantees as in hospital. Likewise, respiratory failure that is corrected with supplemental oxygen and does not generate respiratory work can be treated at home. On the other hand, low-risk patients (PSI I-III, CURB-65 < 2) with additional admission criteria can be treated through HaH, especially those in whom oral antibiotic treatment is not indicated or not possible and parenteral treatment is required. At the other extreme, high-risk patients (PSI IV-V, CURB-65 ≥ 2) without additional risk factors for poor outcome may also benefit from HaH [108]. Overall, it is estimated that 38–48% of CAP patients seen in the ED could be treated at HaH. In all cases, an observation period in the ED of 12–24 h to check clinical and laboratory progress and response to treatment may facilitate decision making.

Criteria for early discharge with subsequent follow-up by HaH teams of patients admitted with CAP do not exist either. In this population group, what justifies HaH intervention is the need to manage comorbidities, prolong treatment due to complications arising during conventional hospitalisation, doubts about adherence to treatment, or the presence of risk factors for readmission [109,110].

One of the procedures that greatly facilitates the treatment of CAP in HaH is the possibility of intravenous antibiotics [111]. To avoid overuse, parenteral antimicrobial therapy should be reserved only for cases with (1) need to rapidly achieve high concentrations of the drug at the site of infection; (2) need for an antimicrobial available only parenterally; (3) inability for oral intake or reasonable doubts about adherence to treatment; (4) absence of a functional gastrointestinal tract or inability to ensure drug absorption; and (5) infection at a site that is unlikely to be treatable with the concentrations of a chosen antibiotic available after oral administration.

The challenge of outpatient parenteral antibiotic therapy (OPAT) is to deliver drugs at the required doses and frequency. Today, elastomeric pumps and programmable electronic devices allow the use of a wide variety of drugs and infusion modalities, including continuous and extended administration of antibiotics. The main limitation in these cases and in intermittent infusions with a schedule of more than 2–3 doses per day is the lack of stability of dilutions at room temperature. Therefore, there is a growing number of studies on the stability of antimicrobials for outpatient use [112,113]. The socio-familial circumstances of patients may also be a limiting factor [114].

As in any healthcare setting, it is necessary to follow clinical practice guidelines and recommendations on antibiotic stewardship programs (ASPs) in CAP patients treated through HaH [115]. Simplification of treatment with easy-to-use antibiotics (usually those administered once daily) should be avoided if they are not part of the treatment alternatives, as well as unnecessary prolongation of intravenous treatment, with both being risks associated with OPAT [116]. In this sense, sequential therapy is indicated when clinical stability has been achieved, temperature, blood pressure, heart rate, respiratory rate, and oxygen saturation has been normalized and peak C-reactive protein has been reduced by more than 50%. Faced with the challenges of appropriate antimicrobial use in HaH, this modality of care also represents an opportunity to improve ASPs in the hospital setting, especially when prolonged treatment is required in patients with complicated CAP (pleural effusion, empyema, multilobar pneumonia), in infections with multidrug-resistant microorganisms, or when sequential therapy is not possible [117].

Main antibiotics and doses used in the treatment of community-acquired pneumonia in hospital at home units [118].

Ceftriaxone 1–2 g/qd/iv, azithromycin 500 mg/qd/iv, and levofloxacin 500 mg/qd/iv are antibiotics that can be administered during the nursing visit or with minimal caregiver collaboration to disconnect the venous catheter after infusion. Amoxicillin/clavulanic acid 1 g/125 mg/tid/iv, due to its short dilute stability (60 min), requires caregiver involvement for dilution and drug administration. In cases of bronchoaspiration or suspected anaerobic infection, ertapenem 1 g/qd/iv or clindamycin 600 mg/qid/iv can be used as an alternative to amoxicillin-clavulanic acid. Against *Pseudomonas aeruginosa* infections, piperacillin-tazobactam 4 g/0.5 g/tid-qid/iv can be administered as a continuous, extended, or intermittent infusion with an electronic infusion pump. The stability of cefepime varies according to concentration, and meropenem 1–2 g/tid/iv should be administered by maintaining the dilution at 2–8 °C or by self-administration. Ceftolozane-tazobactam 1 g/0.5 g–2 g/1 g/tid/iv is stable at 25 °C and ceftazidime/avibactam 2 g/0.5 g/tid/iv should be kept refrigerated to maintain stability for 24 h and requires a prolonged infusion time. For Gram-positive infections, Vancomycin 15–20 mg/kg/bid/iv, Linezolid 600 mg/bid/iv, and Ceftobiprole 500 mg/tid/iv are stable at 25 °C and can be administered by electronic infusion pump. Ceftaroline 600 mg/bid/iv is stable for 24 h at 25 °C and at a concentration of 6 mg/mL. At higher concentrations or higher temperatures, stability varies between 6–12 h, so for administration in HaH it would be necessary to keep the dilution refrigerated (it is stable 24 h at 2–8 °C), perform two nursing visits or resorting to self-administration.

### 3.8. Factors for Readmission in CAP

In Spain, between 22–60% of patients with community-acquired pneumonia (CAP) are admitted from the hospital emergency department (ED) and, after an average stay of 7–10 days, are discharged [119]. Of these, approximately 10–35% require further reassessment by primary care, ED, or even readmission [120,121,122,123] during the following 30 days. Currently, readmission is considered “an adverse outcome” and a relevant “quality care indicator” in patients admitted with CAP. In this regard, factors related to readmission have been differentiated into those derived from the evolution of CAP and those related to other causes, such as comorbidities, social factors, etc. [119].

A systematic review by Prescott et al. [122], which included 12 studies from the United States, Spain, Canada, Croatia, and Sweden, confirmed that 30-day all-cause readmission rates ranged from 16.8% to 20.1% (higher in the US studies and in patients ≥ 65 years). Pneumonia (29.1%), heart failure/cardiovascular causes (22.3%), and chronic obstructive pulmonary disease/pulmonary causes (16.9%) were the three most common reasons for early (30-day) readmission. On the other hand, cumulative readmission rates at 6 months were 35%, 46% at 12 months, and 72% after a median of 3.8 years of follow-up. In another systematic review, Calvillo-King et al. [123] evaluated in 20 studies and examined the impact of social factors related to readmission in CAP, finding that the elderly (≥65 years), those with low educational levels, patients experiencing unemployment, or patients from lower incomes and non-white rpatients had higher rates of readmission after discharge for a CAP process. No associations were found between patients with urban and rural domicile, while sex and living in residences had different results according to studies. Table 6 shows the main causes of readmission after CAP [110,121,124,125,126,127,128,129,130]. The median time to readmission was 8–12 days. During the first three days, 15% of all readmissions occurred, during the first week 33–43%, and 63–66% in the first 14 days [125,126,129]. In total, 95.1% of patients were readmitted once, 4.3% twice, and 0.6% ≥3 times [129].

The factors related to readmission in CAP in the different studies reviewed [121,122,123,124] could be classified into those related to the evolution of CAP itself and those unrelated. The former would include treatment failure, which would include inadequate antimicrobial therapy [119,120,132,133]. Treatment failure is known as clinical deterioration during hospitalization or convalescence with any of the following: radiological progression or complication (involvement of more lobes, cavitation, effusion, empyema, atelectasis, pneumothorax), respiratory failure, need for mechanical ventilation, hemodynamic instability, or development of a new focus of infection. Inappropriate initial antibiotic therapy is defined by the lack of antibiogram-proven sensitivity to the prescribed empirical antibiotic or that which does not conform to the recommendations of clinical practice guidelines.

The most important cause of readmission unrelated to the CAP itself is comorbidity destabilization [120,131,134], generally with Charlson indices ≥2. Among the most important reasons for this destabilization are cardiovascular disorders (congestive heart failure, arrhythmias such as atrial fibrillation and supraventricular tachycardia, acute myocardial infarction, cardiomyopathy, thromboembolic disease, acute arterial ischemia), pulmonary functional or structural alteration (exacerbation of COPD, asthma, pulmonary thromboembolism), deterioration of renal function (acute or chronic acute renal failure), and gastrointestinal disorders (gastrointestinal bleeding, enterocolitis/acute diarrhea associated or not with *Clostridium difficile*, pancreatitis, hepatitis) or endocrine-metabolic disorders (complicated diabetes, ionic disorders, hyperglycemia). Any clinical disorder is susceptible to destabilization in the context of a CAP. Lastly, social problems are included (loneliness, caregiver’s claudication, etc.).

Ensuring clinical stability in CAP has an impact on readmission. Clinical instability is defined as the presence of any of the modified criteria of Halm et al. (body temperature above 37.8 °C, respiratory rate greater than 24 breaths/min, heart rate greater than 100 beats/min, systolic blood pressure less than or equal to 90 mmHg, oxygen saturation less than 90% and/or PaO_2_ less than 60 mm Hg, inability to tolerate oral intake, or altered mental status) [131]. Patients with one criterion of instability could be followed closely at home in a Home Hospitalization Unit. Those with two or more criteria described above should remain hospitalized [120,131].

### 3.9. Therapeutic Failure and Rescue in CAP

There are few studies evaluating the definition and causes of treatment failure (TF) in CAP [135]. Treatment failure has been defined according to different parameters, including symptoms (altered mental status, dyspnea), vital signs (respiratory rate, fever, oxygen saturation), laboratory (white blood cells, biomarkers such as procalcitonin or C-reactive protein, arterial oxygen partial pressure) and radiological findings, and the need for invasive procedures such as BAL or changes in antibiotic treatment. From an academic point of view, TF has been defined as early if it occurs within 1–3 days of hospital admission and late if it develops within 4–7 days of hospitalization [136], so it is critical to identify the causes of TF in CAP in order to improve patient outcomes. The causes to be investigated may be related to the host, the pharmacological treatment, and the pathogen, including the severity of the initial illness, the patient’s age, the occurrence of new or previous decompensated comorbidities, the presence of complications arising from CAP such as pleural effusion or empyema, characteristics of the pathogen itself (resistance patterns), and the activity of the antibiotic treatment used against the pathogen [3].

It is estimated that TF in patients with CAP occurs between 10–15%, and, of those patients who die with CAP, more than 40% have TF. An interesting review that included more than 80 studies identified TF in 16% of patients, and the main reason identified for the cases (30%) was due to detected side effects; only 6% were due to inappropriate empirical antimicrobial therapy (EAT) due to resistant pathogens [137,138].

We have to know if our EAT has been appropriate according to microbiological parameters and/or adequate (according to time of onset, adjusted to PK/PD parameters). Therefore, if we have an inappropriate treatment according to the antibiogram the risk of failure is very high. However, if we prescribe a delayed EAT, we do not consider its correct dosage, its form of administration, its pulmonary penetration capacity, or the presence of organic dysfunctions (such as shock, renal, or hepatic failure), and we can have an appropriate but inadequate EAT, which is also a very important cause of TF.

In general, there are no clinical trials demonstrating better outcomes in CAP using different antibiotics. Although a study with more than 3900 patients identified a higher rate of TF with azithromycin than with levofloxacin [139], a more recent clinical trial observed no difference in the rate of TF between macrolides and quinolones [96]. However, the two most recent guidelines recommend starting empiric antimicrobial therapy (EAT) in hospitalized patients with severe CAP, combining a beta-lactam plus a macrolide and not with quinolones due to the greater efficacy of the former combination than the latter [31,140]. It is true that these guidelines are based on observational studies since they do not include clinical trials indicating differences and that these are necessary to confirm these findings. However, important studies describe significantly lower mortality and less need for IMV with the beta-lactam plus macrolide combination [140].

Inappropriate coverage of EAT can be due to drug-resistant pathogens (DRP) or viral etiology. Although the percentage of CAP due to DRP such as *Pseudomonas aeruginosa*, methicillin-resistant *Staphylococcus aureus* (MRSA), ESBL-producing *Enterobacteriaceae*, *Acinetobacter* spp., and *S. maltophilia* is usually low in most patients (between 1–5%), it is true that, in different subgroups of patients, these figures may be higher, for example, in immunosuppressed patients, those who have received antibiotics, or in certain geographical areas [96,141]. Early microbiological diagnosis with valid respiratory samples collected, if possible, for Gram staining and molecular diagnosis will be important to avoid inappropriate EAT [31,140].

Martin-Loeches et al. suggest using validated prediction clinical scores based on local epidemiology and previous colonization such as “PES” score to avoid DRP and TF [69,140,141]. This score is a predictive model of whether pneumonia is produced *Pseudomonas aeruginosa*, extended-spectrum-betalactamase-producing *Enterobacteriaceae*, or methicillin-resistant *Staphylococcus aureus*. The score assesses factors such as age (<40 = 0 pts, 40–65 = 1 pts and > 65 = 2 pts), sex (Male = 1 pts), previous antibiotic use (2 pts), Chronic respiratory disorder (COPD plus bronchiectasis) = 2 pts, Chronic renal disease = 3 pts, Impaired consciousness in emergency = 2 pts, Fever = −1 pts. For a PES score over 5, the sensitivity, specificity, negative and positive predictive values, and negative and positive likelihood ratios were 36%, 83%, 96%, 11%, 0.77, and 2.09, respectively. These scores are characterized by high negative predictive values (mostly more than 90%), suggesting that their use may allow us to rule out patients who need broad-spectrum empiric antibiotic treatment. Therefore, the use of validated scoring systems together with the data about mucosal colonization and prior antibiotic use can help us to guide appropriate empirical antibiotic treatment in patients with CAP caused by DRP and avoid TF.

#### CAP and Drug-Resistant Pathogens

The incidence of the different drug-resistant pathogens (DRP) in CAP is variable in different studies, ranging from 3.5% to 45% [141,142,143,144,145,146]. Several factors play a role, such as the year or location of the study, patient profiles, their risk factors, severity at presentation, etc. (Table 7) [141,142,143,144,145,146]. The prevalence of DRP-CAP varies according to the region. In the study by Restrepo et al., the continental prevalence of antibiotic-resistant PA-CAP was: 2.5% in North America, 2.2% in Asia, 1.6% in Europe, 3% in South America, and 3.9% in Africa [145].

As can be seen in the table, many risk factors are common to the different pathogens indicated. There is still little information on the actual prevalence of these risk factors specifically [142,144]. The two best known DRPs, because they are the most frequent, are MARSA and DRP-PA. However, as with other infectious diseases, we do not have specific risk factors for the different DRPs causing CAP. Although we should consider an empirical antibiotic therapy (AET) for DTP in patients with a combination of certain risk factors such as those with chronic lung disease with recent antibiotic or steroid use, immunosuppression, receiving chronic invasive treatments or with close contact with home services [141,142,143,144]. The weight of each risk factor for a given pathogen is not equal. For example, the rate of PA-CAP is generally 2–4%, but this can rise to 67% in patients with a previous PA colonization/infection in a patient with severe chronic lung disease [145,146].

The importance of knowing the risk factors for DRP lies in the moment of considering an AET. To propose an AET against DRP generates a radical change in antibiotic therapy in CAP, where most of the time we administer low-spectrum antibiotics such as third generation cephalosporin or macrolides [141,144]. There are different validated scores to try to identify patients at potential risk of DRP to help in decision making at the time of initiating a TSA that is as appropriate as possible [141,142,146].

Salvage treatment will depend on clinical evolution and/or microbiological information. If the TF is due to DRP or as a co-infection, we will have to change or associate some antibacterial and/or antiviral to my EAT. Another possibility is that we may have to adjust the dose of the antibiotic(s) of our initial EAT, for example, because of the development of renal or hepatic failure. We should also consider TF if out EAT causes side effects or drug-drug interactions.

### 3.10. Prevention and Vaccination against Community Acquired Pneumonia (CAP): Immunocompetent and Immunocompromised Host

CAP is fundamentally caused by viruses against many of which there are no vaccines, followed by other microorganisms against which there are vaccines mainly addressed to capsulated bacteria. The importance and recommendations for use differ according to age and pre-existing medical conditions, and those recommendations are more extensive for immunocompromised patients.

#### 3.10.1. Active Immunoprophylaxis with Approved Viral Vaccines

Influenza. Currently, for all viruses causing CAP, active immunoprophylaxis is available against influenza virus, SARS-CoV-2, and varicella-zoster virus. Several types of influenza vaccines are available for the adult and children populations. According to the topic relevance of this chapter, inactivated influenza vaccines (IIV) and live attenuated vaccines (LAIV) can be considered.

WHO and ECDC recommend one dose of influenza vaccine annually for older adults and all persons (over six months of age) with chronic medical conditions [147,148]. In Spain, Interterritorial Council of the National Health System (CISNS) recommends an annual dose for those 65 years and over, for all healthy children six month to <5 yrs, and for Health Care Workers and people who care for institutionalized individuals. People with added risk who should be vaccinated are pregnant women in any trimester of gestation, institutionalized people of any age, adults with chronic cardiovascular, neurological or respiratory diseases, liver diseases, cancer, diabetes mellitus, morbid obesity, etc. Students practicing in health care centers are also encouraged to be vaccinated [149].

Vaccination among the immunocompromised of the above target population should be done with inactivated influenza vaccines (IIV). For adults for whose age group the vaccine is licensed, a quadrivalent vaccine containing two strains of type A influenza virus (H1N1pdm09 and H3N2) and two of influenza B type (Victoria and Yamagata lineages), either adjuvanted, high-dose antigen, or recombinant, are recommended [149,150]. At the time of writing this article, WHO has not included, for the first time, a strain of influenza B virus of the Yamagata lineage in the annual composition of influenza vaccines for the Southern Hemisphere [151]. If this modification is confirmed for the next composition of those in the Northern Hemisphere, it would open new possibilities for antigenic targets in future seasonal influenza vaccines (e.g., two clades of the H3 subtype, neuraminids, etc.), though commenting on this would exceed the limits of this review.

For vaccination of immunocompetent children and adolescents belonging to the age range for which each vaccine is licensed, either quadrivalent IIV or LAIV can be administered. However, LAIV cannot be administered to immunocompromised toddlers and adolescents from 2 to 17 years and is not approved in Spain for other ages over 17.

For children between six months and eight years of age who have never received doses of influenza vaccine before, it is recommended that they receive two doses four weeks apart. A single full dose is recommended for children younger than 9 years old who have been vaccinated in previous influenza seasons and for everyone, regardless of age. Influenza vaccine should be administered in October–November for those living in the Northern Hemisphere. Vaccination is indicated until the end of the annual influenza season for those who did not receive the vaccine in October–November [149,150].

SARS-CoV-2 (COVID19 causing virus). At present, among all the vaccines marketed against SARS-CoV-2, priority is given to the monovalent mRNA vaccine adapted to the omicron lineage (XBB.1.5) [152,153]. The administration of a booster dose against COVID-19 is recommended for the population aged 60 years and older, for institutionalized people in nursing homes and other disability centers, and for those at risk of contracting SARS-CoV-2 and transmitting it to others, including health care workers and health providers. Certain people younger than 60 years should also receive a booster dose, including pregnant women in any trimester of gestation, institutionalized people of any age, and adults with chronic cardiovascular, neurological, or respiratory diseases, as well as those with liver diseases, cancer, diabetes mellitus, and morbid obesity [154]. An immunocompromised host usually needs 3–4 doses to achieve a sufficient level of protection, although nearly 35% of moderate-severe cases may not become protected after 4 doses of COVID-19 vaccine. In those cases, passive immune prophylaxis is recommended if there are mAbs of extended duration that are protective against current circulating variants of SARS-CoV-2.

Varicella-zoster virus. Severe varicella-zoster virus CAP is responsible for an acute pulmonary involvement associated with a significant morbidity and mortality [155]. For varicella prevention, it is widely agreed that in adults up to 65 years of age without evidence of immunity to varicella, serological detection (IgG) should be performed. Serologic negative individuals should be given two doses of varicella vaccine 4–8 weeks apart. The criteria for evidence of immunity to varicella in the adult population are documentation of vaccination with two doses, history of varicella, history of herpes zoster, or serological confirmation of virus varicella IgG. Being an attenuated vaccine, it is contraindicated in pregnant women and persons with severe immunosuppression [156].

#### 3.10.2. Active Immunoprophylaxis with Approved Bacterial Vaccines

Prevention against encapsulated bacteria causing CAP is focused on vaccination against *Haemophilus influenzae* type b and pneumococcus. *Haemophilus influenzae* type b vaccine is recommended in the adult population only in certain situations such as anatomical or functional asplenia (one dose) and hematopoietic stem cell transplant (three doses starting six months after successful transplant) [157].

Several pneumococcal vaccines are available for adult vaccination, including the 23-serotype polysaccharide (PPSV23v) and the 13-serotype (PCV13v) and 20-serotype (PCV20v) conjugate vaccines. The objective is to improve protection against pneumococcus with a single dose, reducing the burden of care and, therefore, the possibility of better coverage. In Spain, systematic vaccination with PPSV23 with one dose is recommended for persons over 65 years of age and risk groups (with revaccination at five years of age). Chronic cardiovascular and respiratory disease, neurological diseases, chronic liver disease, diabetes mellitus, celiac disease, institutionalized persons. PCV13v vaccine followed by PPSV23v (at least eight weeks) are indicated in the adult population of risk groups, as well as immunodeficiencies, immunosuppressive treatment, asplenia, HIV infection, transplantation, cochlear implantation, liver cirrhosis, and Down syndrome (Table 8) [156,157]. Some autonomous regions such as Castilla y Leon, Catalonia, Murcia, and La Rioja have included the PCV20v vaccine. CDC recommends preferably PCV15v or PCV20v conjugate vaccines in both non-risk adult population and at-risk groups [158].

Vaccines under development for possible recommendation in Spain. Recently a Respiratory Syncytial Virus vaccine (RSVV) has published its effectiveness in preventing infection in pregnant woman and extended protection for their infants. In the coming months other RSVV will be available so CAP prevention guidelines will need to be updated [159,160].

#### 3.10.3. New Trends: The Role of Artificial Intelligence in Managing Community Acquired Pneumonia (CAP)

The advances of artificial intelligence have naturally been applied in CAP to improve different aspects of management: hospitalization and mortality risk assessment, prediction of complications (ARDS) and antibiotic treatment, differential etiological diagnosis. A Chest X-ray-based artificial intelligence (AI) model was applied by Quah et al. [161] to generate a mortality risk score based on chest X-ray (CAPE score) that showed a similar prediction capacity (AUC) to traditional PSI and CURB-65 scores but that could improve their discrimination when combined. Similar data were obtained by a validate a causal probabilistic network (CPN) based on clinical data [162].

An artificial neural network model was used by Mo et al. [163] to predict the risk of ARDS in CAP patients based on clinical and laboratory variables; this model based on artificial neural network model has good prediction ability (AUC: 0.977; 95% CI:0.956–1.000), which can be used to calculate the accuracy of ARDS in CAP patients, and specific preventive measures can be given. A machine learning tool was applied by König et al. (CAPNETZ study) to support clinicians in the decision of adding macrolides for treatment of moderately severe CAP to expand the coverage to atypical pathogens and attenuate pulmonary inflammation [164]. This large study (4898 patients) was able to reduce 180-day mortality rate by 27% in comparison to standard of care.

A deep learning-based AI model was created by used by different authors to help physicians in distinguishing COVID-19 from other causes of CAP based on CT scans [165,166,167] or chest X-ray [168]. The support of AI was able to clearly increase diagnostic accuracy of pulmonologists in diagnosing COVID-19 pneumonia. A good diagnostic accuracy was also shown by Han et al., in distinguishing active pulmonary tuberculosis from CAP through a convolutional neural network (CNN) model based on chest X-ray [169].

Other AI-based algorithms were also tested to distinguish between viral and bacterial etiology of CAP based on clinical parameters and chest X-ray, with contrasting results [170] although better results seem to be available in pediatric pneumonia [171,172].

## 4. Conclusions

CAP represents the most important cause of mortality due to infection in industrialized countries. Although diagnostic and treatment guidelines are in place internationally, information has recently emerged that would help in optimizing its management. The etiological vision is beginning to change thanks to the progressive introduction of syndromic platforms based on real-time PCR techniques that demonstrate the majority participation of viruses as the main etiological agent or at least in coinfection. This constitutes a new challenge, both for the pathogenic interpretation of the syndrome and for therapeutic management in the world of stewardship. The choice of therapy in CAP requires a risk-benefit assessment for each individual patient, taking into account local epidemiological data, individual risk factors, as well as documented antibiotic allergy. The combination of a beta-lactam with a macrolide seems to be the most recommended starting strategy, with a duration of 5 to 7 days. The treatment of hypoxemia should be balanced with the risk of excessive oxygen supply and should be adapted to the clinical response and the rest of the therapeutic strategy. Similarly, steroid treatment must also be individualized, since it has proven to be useful in distress and shock situations but has not benefited all clinical profiles in the case of COVID and may have a negative impact on the immunological control of the infection. Continuity of care in HaH units is the most important challenge, especially in patients in whom, due to comorbidity or intolerance, optimal defervescence cannot be guaranteed in sequential therapy. Administering intravenous drugs at the required doses and frequency and monitoring comorbidity destabilization are critical to avoid readmission. The most frequent causes of therapeutic failure in CAP are the presence of resistant pathogens, inadequate concentration of antimicrobials in the focus and the severity of the pneumonic process that often destabilizes comorbidity. Vaccination is the measure with the greatest impact in reducing the incidence and mortality of pneumonia, both in immunocompetent and immunocompromised patients.

## Figures and Tables

**Table 1:** Microbiological findings in patients with community acquired pneumonia using different laboratory techniques refs [2,11,12].

Pathogens	% of Positive Cases with		
	Bundels of Methods (Gadsby-2021) (2011–2014)	Multiplex Syndromic Pannels	
		Pre-COVID-19 (Ref. Serigstad-2022 and Serigstad-2022) (2019–2020)	COVID-19 (Ref. Serigstad-2022b) (2020–2021)
Bacteria *	15–30	62	71
*S. pneumoniae*	5–16 **	15–25	15
*H. influenzae*	<1–7	22–36	26
*S. aureus*	2–5	3–7	23
*S. agalactiae*	-	4–6	6
*S. pyogenes*	-	0	1
*M. catarrhalis*	-	7–11	10
*Pseudomonas* spp.	<1–3	2–3	3
*E. coli*	-	4–8	4
*K. pneumoniae*	-	3	1
*S. marcescens*	-	3	1
*Proteus* spp.	-	2	1
*K. oxytoca*	-	1	1
*K. varicola*	-	0	3
*Acinetobacter* spp.	-	1	0
*E. cloacae* complex	-	1	3
*Legionella pneumophila*	1	1	0
*Mycoplasma*/*Chlamydia*	<3–1	2	0
Mycobacteria	<1–2	-	-
Nocardia	0–1	-	-
Fungy *	1–3	4	11
*C. albicans*	ND	0–3	10
*P. jirovecii*	ND	0–1	1
Virus *	3–27	30–36	21
Rhinovoirus	0–12	1–3	15
Coronavirus (229E, OC43, HKU1, NL63)	0–3	3–5	0
SARS-CoV-2	-	-	13
Metapenumovirus	0–4	9–17	0
Influenza	1–3	14–30	0
Parainfluenza	2–3	2	1
VRS	2–3	3–7	0

* Percentage of patients; ** data are presented as percentage of total cases studied (total might exceed 100% due to coinfections).

**Table 2:** Radiological findings in different types of pneumonia.

Chest X-ray		
Virus	Typical Bacteria	Atypical Bacteria
Bilateral	Unilateral	Asymmetric
Central	Consolidation Bronchopneumonia	Non-homogeneus consolidation
Symmetric	Asymmetric	Alveolo-interstitial pattern
Interstitial ground-glass opacity	Alveolar pattern	
**Computed tomography**		
**Virus**	**Typical Bacteria**	**Atypical Bacteria**
Ground-glass opacities Centrilobular nodules Bronchial wall thickening Bilateral	Consolidation Lobar pneumonia Bronchogram	Ground-glass opacities Bronchovascular bundle thickening Reticular or linear opacities Unilateral
**Lung ultrasound**		
**Virus**	**Bacteria**	**Both**
Alveolar-interstitial pattern	Consolidation: predominantly subpleural hypoechoic region or a hypoechoic region with liver-like density with usually irregular, non-rounded borders	B-lines: perpendicular to the pleural line and parallel to each other. Usually caused by decreased alveolar aeration and fluid accumulation under the visceral pleural, thickening of interlobular septa, mostly related to interstitial occupation
Combined with preserved areas	Air bronchogram: hyperechogenic tree-like images corresponding to air-filled bronchi	Often seen focally, multifocally or patchily in ground-glass opacities or around areas of consolidation

**Table 3:** Diagnostic stewardship in community-acquired pneumonia. Based on references [34,35,36] and on the authors’ own experience.

Setting	Microbiological Tests	Recommendation
Primary care, outpatient clinic, long-term facilities	-Rapid antigen-antigen-based diagnostic tests for virus	-Not recommended with the exception of SARS-CoV-2
	-Sputum culture	-Not recommended
	-Blood culture	-Not recommended
	-Molecular tests for detection of bacterial and viral pathogens	-Not recommended
	-Use of CRP/procalcitonin	-Not recommended
Emergency department	Rapid antigen-antigen-based diagnostic tests for virus	Not recommended with the exception of SARS-CoV-2
	Gram stain and culture of respiratory secretions.	Recommended in patients with severe disease, immunocompromised patients, and in inpatients empirically treated for MRSA or *P. aeruginosa*
	Blood culture	Recommended in patients with severe disease, immunocompromised patients, and in inpatients empirically treated for MRSA or *P. aeruginosa*
	Nares screening for MRSA	Recommended in inpatients empirically treated for MRSA.
	Urinary antigen test (*S. pneumoniae* and *Legionella pneumophila*)	Recommended in patients with severe CAP and in those with epidemiological risk factors for *L. pneumophila* infection
	Molecular tests for detection of bacterial and viral pathogens	Recommended in patients with severe CAP for the detection of influenza viruses and SARS-CoV-in immunocompromised patients. Recommended detection of influenza viruses in periods of high influenza activity Rapid respiratory syndromic panels may be considered in certain patients
	Use of procalcitonin	Not recommended to determine initiation of antibacterial therapy

**Table 4:** ATS/IDSA intensive care unit admission criteria. The presence of one major criterion or ≥3 minor criteria imply admission in ICU.

Major Criteria Invasive Mechanical Ventilation Septic Shock with the Need for Vasopressors
Minor criteria Respiratory rate ≥ 30 breaths/min PaO_2_/FiO_2_ ≤ 250 Multilobar infiltrates Confusion/disorientation Uremia (BUN level ≥ 20 mg/dL) Leukopenia (WBC count < 4000 cells/mm^3^) Trombocytopenia (platelet count < 100,000 cells/mm^3^) Hypothermia (temperature < 36 °C) Hypotension requiring aggressive fluid resuscitation

**Table 5:** Initial Treatment Strategies for patients with Community-acquired Pneumonia [62]. * The duration of treatment should be individualized according to clinical stability with a minimum of 5 days. ** Risk factors include prior respiratory isolation of MRSA or *P. aeruginosa*, severe COPD, bronchiectasis, or recent hospitalization and receipt of parenteral antibiotics (in the last 3 months).

Primary Care Regimen *	Hospital Admission Regimen *	ICU Admission Regimen
Oral amoxicillin 1 g/8 h or oral amoxicillin-clavulanic 875/125 mg/8 h (if asthma or COPD) or cefditoren 400 mg/12 h (alternative) Plus Macrolide (oral azithromycin 500 mg/24 h/3 days or clarithromycin 500 mg/12 h) Or Levofloxacin 500 mg/12 h (1–2 days) and then 500 mg/24 h or Moxifloxacin 400 mg/24 h	Ceftriaxone 2 g/24 h iv or cefotaxime 2 g/8 h iv or ceftaroline 600 mg/12 h iv (if post-influenza pneumonia or risk of *S. aureus*) Plus Oral/iv macrolide (azithromycin 500 mg/24 h/3 days or clarithromycin 500 mg/12 h) Or Levofloxacin 500 mg/12 h iv (1–2 days) and then 500 mg/24 h or moxifloxacin 400 mg/24 h iv	Ceftriaxone 2 g/24 h iv or cefotaxime 2 g/8 h iv or ceftaroline 600 mg/12 h iv Plus Macrolide (azithromycin 500 mg/24 h iv or clarithromycin 500 mg/12 h iv) or quinolone (levofloxacin 500 mg/12 h or moxifloxacin 400 mg/24 h)
		If PES score ≥ 5 or previous MDR colonization **: Meropenem 1 g/8 h iv + Levofloxacin 500 mg/12 h iv + Ceftaroline 600 mg/12 h iv or Linezolid 600 mg/12 h iv

**Table 6:** Main risk factors related to readmission in CAP.

Reference	Type of Study	30 Days Readmission *n* (%)	CAP Related Factors	Non-CAP Related Factors	Commentaries
Adamuz J, et al. [121]	Prospective Single centre	*n* = 934 (25.5%)	Worsening signs and symptoms of CAP: 38.9%	CAP non-related: 43% Cardiovascular (16.5%), pulmonary disease (11.1%), Neoplasia (6.9%), other infections (2.8%), Neurologic disease (1.4%), gastrointestinal disease (1.4%), treatment toxicity (1.4%). Admission within the previous 90 days: OR 2.47; (CI 95%: 1.11–5.52) comorbidities: OR 3.99; (CI 95%: 1.12–14.23)	In 18% it was attributed to the addition of CAP related and non-related
Capelastegui, et al. [124]	Prospective Single centre	*n* = 1.117(7.3%)	Worsening signs and symptoms of CAP: 35.8% Treatment failure: HR 2.9; CI 95%: 1.2–6.8 Clinical instability at discharge: HR 2.8; CI 95%: 1.3–6.2 Both factors: HR 9.0; CI 95%: 3.2–25.3	CAP non-related: 64.2% age ≥ 65: (HR: 4.5; CI 95%: 1.4–14.7) Charlson Comorbidity Score over 2: (HR: 1.9; CI 95%: 1.0–3.4) Both factors: (HR, 5.3; CI del 95%, 1.6 a 18.1)	A greater number of factors was associated with a higher risk of readmission (*p* < 0.001). At hospital discharge, patients without risk factors had a probability of readmission of less than 1.5%.
Jasti, et al. [125]	Prospective Multicentric	*n* = 577 12%	CAP related: 20%	CAP non-related: 74% Coronary heart disease: OR: 2.7 (CI 95%: 1.5–4.7) COPD: OR: 2.3 (CI 95%: 1.3–4.1)	Social factors: Basic education OR: 2 (CI 95%: 1.1–3.4). Unemployment OR: 3.7 (CI 95%: 1.1–12.3)
Chakrabarti, et al. [126]	Prospective Multicentric	*n* = 12.157 (26%)	No records	Non-metastatic cancer: OR: 1.7 (CI 95%: 1.3–1.7) Complicated diabetes: OR: 1.6 (CI 95%: 1.2–1.8) Chronic kidney disease: OR: 1.2 (CI 95%: 1.1–1.3) Dementia: OR: 0.8 (CI 95%: 0.6–0.9)	Those readmitted within 14 days were more likely to have metastatic cancer (6.6% vs. 4.5%; *p* = 0.03) compared to those readmitted at 15–30 days.
Toledo, et al. [110]	Prospective Multicentric	*n* = 1756 over 65 (11.4%)	CAP related: 44.5%	CAP non-related: 50%. Living with a person aged under 15—OR: 2.10 (95% CI 1.01 to 4.41) ≥3 hospital admissions in previous 90 days—OR: 1.53 (95% CI 1.01 to 2.34) Chronic respiratory failure—OR: 1.74 (95% CI 1.24 to 2.45) Heart failure—OR: 1.69 (95% CI 1.21 to 2.35) Chronic liver disease—OR: 2.27 (95% CI 1.20 to 4.31) discharge to hospital at home unit—OR: 5.61 (95% CI 1.70 to 18.50)	No associations were found with seasonal influenza or pneumococcal vaccination in any of the three previous seasons.
Jang, et al. [127]	Retrospective Multicentric	*n* = 862 (8.4%)	CAP related: 37.5% clinical instability at discharge (≥1)—HR: 5.3; (CI 95%: 2.2–13.2) PSI ≥ 4 —HR: 10; (CI 95%: 1.4–75.5)	CAP non-related: 62.5%. Chronic kidney disease: OR: 5.7 (95% CI, 2.2–14.7) Chronic lung disease: OR: 2.8 (95% CI: 1.3–5.9)	CAP related and non-related in 16%
Mather, et al. [128]	Retrospective Multicentric	*n* = 965 (15.5%)	CAP related: 16.9% Hematocrit below 30% Leucocytosis over 12,000 mm^3^	Male sex (OR 1.59, 95% CI 1.03–2.45) 3 or more previous admissions (OR 1.84, 95% CI 1.22–2.78) COPD (OR 1.63, 95% CI 1.07 to 2.48) Cancer (OR 2.18, 95% CI 1.24 to 3.84) Median income under <$43,000 (OR 1.82, 95% CI 1.18 to 2.81) History of anxiety or depression (OR 1.62, 95% CI 1.04 to 2.52) Hematocrit under 30% (OR 1.86, 95% CI 1.07 to 3.22)	This study analyses mainly non-related CAP
Nguyen, et al. [130]	Retrospective Single centre	*n* = 582 (11.9%)	CAP related: 43.5% Multidrug-resistant bacteria OR: 2.6 (IC 95%: 1.1–6.6)	Charlson Comorbidity Score ≥ 3 OR: 1.4 (CI 95%: 1.1–1.8) Most frequent comorbidities:Cardiovascular, Chronic kidney disease,	The only study in which CAP related are the most frequent.
Halm, et al. [131]	Prospective Single centre	*n* = 680 (9.9%)	clinical instability at discharge (=1) OR: 1.6; (IC95%, 1.0–2.8) clinical instability at discharge (≥2) OR: 5.4; (IC95%, 1.6–18.4)		19.1% were discharged with 1 or more instabilities. 10.5% of patients with no instabilities were readmitted vs. 13.7% whohad 1 instability or 46.2% of those with ≥2 instabilities (*p* < 0.003)

**Table 7:** PA: *Pseudomonas aeruginosa*; MR: Multi-resistant; Enterobact: *Enterobacteriaceae* (most of studies included extended-sprectum beta-lactamase-producing *klebsiella* spp. or *E coli*—ESBL) refs. [141,142,143,144,145,146].

	Incidence	Risk Factors	Severity of Presentation
MRSA	1.7–24%	Age Sex: Male Diabetes mellitus Immunosuppression COPD/bronchiectasis Cerebrovascular disease Prior antibiotics Poor/low functional status Nursing home residence Admission from long-term care Recent hospitalization (<90 days) Gastric acid suppression Tube feeding Chronic renal disease Hemodialysis Altered mental status MARSA colonization in the previous year Home wound care Ulcer pressure Recurrent skin infection Recent chemotherapy Tobacco use	Bilateral pulmonary infiltrations Vasopressor administration Severe pneumonia Fever Respiratory rate Bacteremia Altered mental status Elevated BUN > 19 mg/dL Invasive mechanical ventilation ICU admission PSI level: IV or V
PA and MR-PA	0.7–18.8%	Age Sex: Male Immunosuppression COPD/bronchiectasis Very severe COPD (FEVi < 30%) Oxygen therapy at home Prior antibiotics (oral/intravenous) Use of steroids (included inhaled) Poor/low functional status Admission from long-term care Recent hospitalization (<90 days) PA previous colonization/infection Heart failure Gastric acid suppression Tube feeding Chronic renal disease Hemodialysis MARSA colonization in the previous year Home wound care Ulcer pressure Recent chemotherapy Prior tracheostomy	Bilateral pulmonary infiltrations Vasopressor administration Fever Respiratory rate Altered mental status Severe CAP Invasive mechanical ventilation PaO_2_/FiO_2_ < 200 Criteria of ARDS Elevated BUN > 19 mg/dL High serum levels of CRP PSI level: IV or V ICU admission
MR Enterobact	0.2–6.9%	Age COPD/bronchiectasis Prior antibiotics Poor/low functional status Admission from long-term care Nursing home Home wound care Recent hospitalization (<90 days) Chronic renal disease Gastric acid suppression Tube feeding Immunosuppression Previous infection by MDR (<1 year) Recent chemotherapy	Bilateral pulmonary infiltrations PaO_2_/FiO_2_ < 300 Invasive mechanical ventilation
*Acinetobacter* spp.	0–1.2%	Age Admission from long-term care Recent hospitalization Home infusion therapy Hemodialysis Immunosuppression Recent chemotherapy	Severe pneumonia

**Table 8:** Pneumococcal vaccination guidelines in Spain. PPSV23v: 23-valent Pneumococcal polysaccharide vaccine. PCV13v: 13-valent pneumococcal conjugate vaccine, PCV20v: 20-valent pneumococcal conjugate vaccine. HIV: Human Immunodeficiency Virus. CSF fistula: cerebrospinal fluid fistula [151,152].

Population Group	Recommended Pattern	Modifications in Autonomous Regions
Over 65 yrs without risk factors	PPSV23v (1 dose)	PCV20v or PCV13v (1 dose)
Over 18 yrs with chronic pathology: chronic cardiovascular and respiratory disease, severe neurological and neuromuscular disease, chronic liver disease, Diabetes mellitus, celiac disease, institutionalized persons	PPSV23v 1 dose + revaccination each 5 years	PCV20v or PCV13v (1 dose)
Over 18 yrs high risk groups: immunodeficiencies and complement system deficiencies, immunosuppressive treatment, asplenia or severe splenic dysfunction, HIV infection, chronic renal failure and nephrotic syndrome, transplant, CSF fistula, cochlear implant, history of invasive pneumococcal disease, liver cirrhosis and chronic alcoholism, Down syndrome.	PCV13v (1 dose) + PPSV23v (1 dose) (at least 8 weeks)	PCV20v (1 dose) + PPSV23v (1 dose) (at least 8 weeks)

## Data Availability

Not applicable.
